# Drug-Resistant *Leishmania tropica* Parasites Detected in Iranian Cutaneous Leishmaniasis

**DOI:** 10.1371/journal.pmed.0030230

**Published:** 2006-04-18

Leishmaniases are parasitic diseases that are endemic (constantly present) in many tropical and temperate countries. Every year, 2 million people become infected with one of 20 pathogenic species of *Leishmania* through the bites of infected female sand flies. These pick up parasites by biting an infected animal (zoonotic transmission) or an infected person (anthroponotic transmission). In their human host, *Leishmania* parasites reproduce inside macrophages—white blood cells that usually kill microorganisms, clear up cellular debris, and activate other immune cells. When the macrophages are full of parasites, they burst—this destruction causes the symptoms associated with leishmaniases—and the released parasites infect further macrophages.

In cutaneous leishmaniasis—the most common form of the disease—patients develop skin ulcers a few weeks after being bitten by infected sand flies. These usually heal spontaneously but leave ugly, sometimes disabling, scars. Cutaneous *Leishmania* infections can spread to the nose or mouth to cause mucocutaneous leishmaniasis, which destroys the sensitive linings of these organs. Cutaneous and mucocutaneous leishmaniases are not life-threatening in themselves, but patients can develop fatal secondary infections. Visceral leishmaniasis, which affects the spleen and other internal organs, is often fatal if untreated.

Leishmaniases are usually treated with pentavalent antimony-containing drugs, such as meglumine antimoniate (Glucantime), but patients are becoming increasingly unresponsive to these drugs. In India, for example, more than 60% of cases of visceral leishmaniasis do not respond to treatment. Unresponsiveness can be caused by the parasite developing drug resistance, by changes in the host's immunological status, or by suboptimal treatment regimens. Ramtin Hadighi, Mehdi Mohebali, Marc Ouellette, and colleagues have been investigating whether the increased incidence of Glucantime-unresponsive cutaneous leishmaniasis in Iran correlates with parasite resistance to the drug. They now report that treatment failure for cutaneous leishmaniasis in Iran, like the treatment failure seen for visceral leishmaniasis in India, is due to Glucantime-resistant parasites.

The researchers isolated *Leishmania* parasites from 185 skin lesions from untreated patients living in Mashhad, a region of Iran where anthroponotic cutaneous leishmania is endemic. Of these patients, 20 did not respond to Glucantime—their skin ulcers failed to heal. To find out if this was due to drug-resistant parasites, the researchers infected mouse macrophages with all 185 isolates and then treated the infected cells with Glucantime. Several days later, the parasites surviving inside the cells were stained with a dye and then counted using a microscope. The researchers report that although initial infection rates were similar, parasites from the unresponsive patients were resistant to intermediate or high levels of Glucantime. On average, parasites from unresponsive patients were 4-fold less susceptible to Glucantime than parasites from responsive patients.

Next, the researchers partly characterized the 20 drug-resistant parasite isolates and 11 drug-susceptible isolates. By sequencing the gene for the metabolic enzyme pteridine reductase 1, the researchers discovered that 28 of the isolates were *L. tropica*; the remaining three were *L. major*. Only one unresponsive isolate was *L. major*; the rest were *L. tropica*. The researchers also used pulsed-field gel electrophoresis to separate and study *Leishmania* chromosomes. Because these evolve quickly, the chromosome composition (karyotype) of different isolates indicates their genetic relatedness. The *L. major* isolates formed one group using this technique but the *L. tropica* isolates fell into three distinct groups, each of which included drug-susceptible isolates and isolates with intermediate and high Glucantime resistance. In other words, susceptible and resistant isolates were often closely related. Finally, the researchers confirmed the drug sensitivity of several closely related strains by testing their ability to grow in a human monocyte cell line in the presence of Glucantime, and also showed that drug resistance was stable over time in resistant isolates but could be reversed by treatment with an inhibitor of glutathione biosynthesis. This last result indicates that thiols (molecules containing a sulphur atom bonded to a hydrogen atom) may be important for the resistant phenotype, and may suggest a way to reverse drug resistance.

Overall, these results provide the first evidence that *Leishmania* parasites can acquire drug resistance that contributes to treatment failure in cutaneous leishmaniasis. They also indicate that Glucantime-resistant *L. tropica* isolates are now frequent in Iran. Additional work is needed to understand the nature of the resistance mechanisms, with the goal to improve diagnosis and treatment of resistant leishmaniasis.

## 

**Figure:**
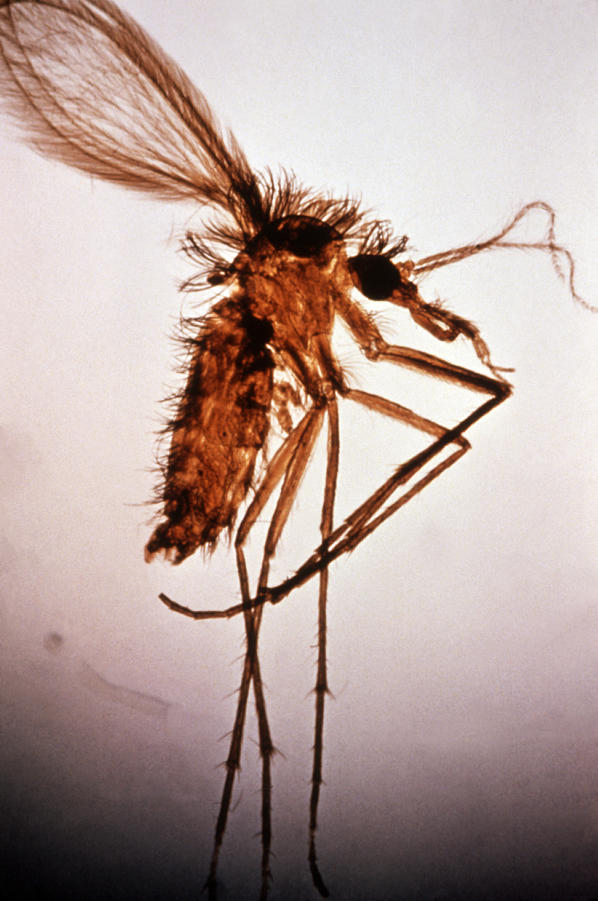
Female sandflies transmit the Leishmania parasite

